# An Anticipatory Geriatric Strategy: To Better Care for Those Americans Not Yet Old

**DOI:** 10.1155/2011/154246

**Published:** 2011-09-29

**Authors:** Neil J. Nusbaum

**Affiliations:** Northampton VA Medical Center, University of Massachusetts, Leeds MA 01053, USA

## Abstract

Current US public policy decisions will have impact on national plans to care for the aging American baby boomer population over the next several decades. The recent health care legislative debate has been largely about the structure of health care for those still too young to be covered by Medicare, but the legislation may have important implications for the average rates of accumulating chronic illness and disability in midlife and influence the care needs for that cohort of individuals even after they become elderly.

## 

As we prepare for the aging of the American baby boomer population, much focus has come to the issue of the expected costs of providing that care, and this issue has figured prominently in the ongoing discussions about implementation of the recent national health care legislation. The future cost of caring for an aging population can be decomposed into three separate factors. The first of these factors, the numbers of middle-aged Americans poised to become elderly in the next several decades, is essentially an immutable quantity. The second factor, the cost of caring for each episode of illness, has received much attention during the health care debates of recent months, with a variety of strategies (such as use of electronic health records to guide care, measures to avoid duplicative laboratory tests, and increased resources to ferret out healthcare fraud) being proposed to make health care delivery more cost effective.

The third factor of the equation, which has gotten much less attention, is the average level of baseline health of the older individual, which in turn drives the average amount of care that needs to be delivered to a given older individual per year. It may be particularly useful to focus on the subset of health care interventions that represents “dominating” strategies; that is, those interventions which produce an improvement in public health at the same time that they also save money. A focus on such dominating strategies can offer an initial opportunity to begin the process of expanding the health care safety net in a relatively painless fashion, to precede the much more challenging task of making cost-benefit decisions about other health care efforts that do not save money but that are cost effective.

The search for dominating strategies will become substantially easier to accomplish as the US Center for Medicare and Medicaid Services (CMS) has recently announced measures to encourage the “meaningful use” nationally of electronic health records through provider incentives under Medicare and Medicaid starting in 2011 [1, 2]. Care for the elderly under Medicare, and for the indigent under Medicaid, target two vulnerable subsets of the U.S. population who often have complex medical care needs. These populations might be especially benefited by initiatives to use their electronic health data to help deliver cost-effective care to them. 

The meaningful electronic health record is intended to ultimately include the BMI, blood pressure, an up-to-date problem list, active medication list, ambulatory quality measures, and some decision support rules among other features. This new electronic record, combined with the current interest in funding health care effectiveness research, should create an ideal environment to promote research efforts that can convincingly identify the dominating healthcare strategies in caring for aging baby boomers.

Adults who lack insurance coverage prior to age of 65, and who thus have an economic disincentive to seek health care in midlife, are likely to be higher users of health care once they become eligible for Medicare coverage [3]. Interventions in late midlife can favorably influence healthcare costs into late life in a variety of ways. Lifestyle interventions that produce durable behavioral change, such as permanent smoking cessation, would have even more health and economic benefits the earlier in life they were implemented. Even if a lifestyle behavioral change in late midlife was only temporarily effective, such as improving adherence to an antihypertensive regimen for a year or two, net benefits could persist into old age (e.g., fewer individuals turning age of 65 being already disabled by a remote prior stroke). Finally, some surgical and other interventions made in late midlife will have intrinsic capability to remain in place after age of 65, even if done long previously. 

National health care policy can benefit from more attention to the process by which individuals transition into Medicare eligibility [4]. Consider modeling the economic and public health effect of Medicare offering to be the secondary payor at age of 63 for individuals who are obese diabetics as indicated by the combination, respectively, of their electronic health record BMI, of diabetes as shown by their diagnosis list, by a medication list containing insulin or other diabetes-specific drugs, and/or by laboratory criteria (hemoglobin A1c and/or blood glucose levels). In particular, one would seek to follow up on the evidence in the recent medical literature [5, 6] suggesting that bariatric surgery is a dominating strategy for management of obese diabetics based even on the savings that accrue within two years.

In this hypothetical example, assume that the actuarial likelihood is that a typical 63-year-old obese diabetic would still have a life expectancy greater than ten years, so that in most cases they would yield two years of savings prior to reaching age 65 (and current Medicare eligibility), and typically greater than eight years of saving for ongoing healthcare during the post-65 period of Medicare eligibility. One might wish to include a modest adjustment to discount to present (when patient's age is 63) value for the savings in the succeeding pre-Medicare and post-Medicare eligibility periods. 

In actual practice, of course, one would anticipate that individuals with obvious life limiting conditions would not be offered or accept bariatric surgery, so that the cost of any such surgical intervention would in clinical practice likely be focused on the relatively healthier among the 63-year-old obese diabetics, those who might have a relatively longer life expectancy. Clinical care decisions that focus expensive surgical interventions on those most likely to derive long-term benefit, in general, would translate at the societal level into increased cost effectiveness of the intervention.

Why have dominating healthcare strategies not been more widely employed? In some cases, this failure represents a lack of knowledge to identify clearly those strategies which in fact are dominating. More research expenditures are now being actively discussed to look at comparative effectiveness of interventions, and such studies should provide much data to identify dominating healthcare strategies. Sound research expenditures that enable more effective use of health care resources, spending millions on research to help us better allocate billions on health care, are too likely to represent economically dominating expenditures.

In some cases, the failure to adopt dominating healthcare strategies may represent a consequence of disparate distribution of costs and benefits, so that a payor for example may incur most of the cost of the health care intervention yet reap only a fraction of the economic benefits. A typical example would occur in a healthcare insurance market where those insured frequently change from one insurer to another, so that the current insurer has a primary economic interest in minimizing current health care expenditures even at the risk that this will lead to worsening future health outcomes and higher future health care expenditures for the patient currently insured by them, since the late future health care expenditures are likely to be external to the current insurer's economic interest. A more global way of avoiding these issues is to move to a single payor system that minimizes such economic externalities. Short of that intervention, one should look for ways to promote dominating healthcare strategies as a societal good, regardless of whether the implementation of the dominating strategy happens to be in a particular economic interest of the specific present insurer. 

If the American health care system should adopt a policy of funding selected preventive health measures via Medicare for those under age of 65, then there is a risk that this may result in cost shifting for care from the current private insurer to Medicare. One strategy to prevent this result might be to allow Medicare to cover payment for select healthcare interventions prior to age of 65, but only as the secondary payor, so that those individuals under age of 65 with private insurance would primarily be covered by their insurance company. Medicare in such cases might absorb the deductible and copay, so that the dominating healthcare intervention would in any event not result in an out-of-pocket cost to the individual consumer. 

Other more complex cost allocation schemes could certainly be considered. One might for example adjust the share to be paid by Medicare to be relatively higher if the intervention was made when the patient was very nearly at the age of Medicare eligibility, or if the patient were of lower income, or if the patient had already incurred significant out-of-pocket healthcare costs that year. The argument from a societal perspective is that dominating healthcare expenditures are worth making, that their total benefit to society is greater than their total cost to society. The decision of how to allocate those costs most fairly is certainly important, but it is separate from and subsidiary to the fact that the costs are worth incurring. 

The strategy proposed here focuses on those individuals approaching age of 65, but it has implications for both older and younger segments of the population. On the one hand, introduction of dominating healthcare strategies such as lifestyle changes in middle age could tend to increase the number of who survive to be among the oldest old [7–9]. This would have the cost effect of increasing the number of years of healthcare coverage required to support a prolonged life expectancy into old age, but this is certainly a cost we should be willing to accept. 

On the other hand, introduction of a limited CMS role to fund dominating healthcare approaches in midlife could, if the country so chooses, later become the foundation for public policy experience for expanding a yet broader role for public support of healthcare to individuals below age of 65. In the current economic environment, one might choose first to publicly fund midlife healthcare interventions that are demonstrated to be dominating and so actually save money in terms of lifetime healthcare costs, but then later expand coverage to those additional midlife healthcare interventions that are very cost effective even though they do involve some net expense. 

Some of the most contentious issues in health care policy today revolve around issues of resource allocation and distributive justice. One point of contention is what fraction of gross domestic product should be devoted to health care. A second issue is how health care expenditures should be allocated across generations; including whether health care needs of the young should be favored over those of the old, in order to maximize the number of life-years gained by society for each dollar of health care expenditure. Yet, a third concern is that national health care costs are growing faster than the rate of inflation and faster than the rate of overall growth in domestic product. 

A focus on dominating healthcare strategies does not resolve any of these three issues, but to some extent it can sidestep each of them. Since an economically dominating strategy by definition is one that saves money over the course of time, that saving should tend to decrease the fraction of gross domestic product spent on health care. Second, interventions that produce long-term gains in health status may be administered to the young, but still produce benefits that persist into old age. Finally, these dominating strategies can help moderate the rate of rise in health care costs. 

I would argue that an effort to fund strategies identified as dominating is useful as an overall approach, even recognizing that some strategies labeled as dominating will in fact prove to have a net cost. Many of these errors are likely to be cases where the prediction of domination was only slightly off the mark, so that the net cost of the strategy will be relatively small. As a result, an effort to fund putatively dominating strategies in practice is likely to direct funding to a mixture of strategies, some of which will in fact be dominating, and most of the rest of which will be highly cost effective.

Calculating whether a health care strategy is dominating may depend on a number of assumptions, not only about health care itself, but also about economics. If a costly health care intervention only produces its savings benefits many years in the future, one might value that result less highly the further in the future the benefit will be realized. Should the value of an intervention that produces long-delayed future savings be discounted to reflect the present value of those savings? If so, what discount rate should be applied; a subjective discount rate chosen by survey, a discount rate based on the long-term interest rate, or some other metric?

For many health care innovations, we may simply lack the data to do complete calculations of the most distant benefits. Consider the case of a new vaccine. The longer the duration of protection from a dose of vaccine, the more likely it is that use of the vaccine will be a dominating strategy. 

At the time of initial approval of an innovative vaccine product, the manufacturer is likely to have short-term data on immunogenicity, safety, and efficacy. There will not probably be enough time to evaluate whether the initial dose offers lifetime protection, or whether the vaccination will need to be repeated in 5–10 years to maintain protective titers. One might try to extrapolate the rate of titer decline at ten years based on the fall in titer in the initial years, but this would be a speculative enterprise. On the other hand, if the protective effect of the vaccine can be shown to yield enough health benefit in the first year or two to fully cover the expense of the vaccine, then vaccination is likely to be a dominating strategy regardless of whether the vaccine protection extends unabated or whether it wanes in subsequent years. 

In a setting of rising health care expenditures and limited resources, there has still been considerable reluctance to confront hard tradeoff situations where high costs are associated with important health care benefits. Efforts to control costs have often looked for easier cases first. These efforts have often taken a sharply negative tone, looking at ways to decrease “fraud, waste, and abuse,” however, the particular speaker may define these terms. These efforts reflect in part an attempt to explore parts of the health care budget where there is no tradeoff, because the expenditure doesn't produce any real benefit. Dominating health care strategies represent the mirror image easy case, a positive opportunity to secure societal health benefit at no net cost. Decision makers should be as diligent in working to maximize the implementation of dominating healthcare strategies as they have been in advocating to minimize fraud, waste, and abuse.

##  Disclaimer

Personal opinions are those of the author, not official.
